# 4-Methoxy­anilinium chloride

**DOI:** 10.1107/S1600536809035429

**Published:** 2009-09-09

**Authors:** Min Min Zhao

**Affiliations:** a Ordered Matter Science Research Center, College of Chemistry and Chemical, Engineering, Southeast University, Nanjing 211189, People’s Republic of China

## Abstract

The crystal structure of the title compound, C_7_H_10_NO^+^·Cl^−^, was synthesized by the reaction of 4-methoxy­aniline and hydro­chloric acid. In the crystal structure, the ions are involved in inter­molecular N—H⋯Cl hydrogen bonds.

## Related literature

For a similar organic acid-base product, see: Wu *et al.* (2006 ▶). This work is part of a systematic investigation of dielectric–ferroelectric materials, including organic ligands, metal–organic coordination compounds and organic–inorganic hybrid materials; see: Li *et al.* (2008 ▶); Hang *et al.* (2009 ▶).
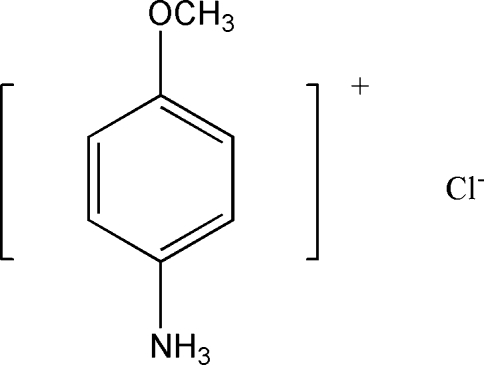


## Experimental

### 

#### Crystal data

C_7_H_10_NO^+^·Cl^−^
*M* *_r_* = 159.61
Orthorhombic, 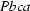

*a* = 8.905 (2) Å
*b* = 8.489 (2) Å
*c* = 21.817 (4) Å
*V* = 1649.3 (6) Å^3^
*Z* = 8
Mo *K*α radiation
μ = 0.40 mm^−1^
*T* = 298 K
0.20 × 0.20 × 0.20 mm

#### Data collection

Rigaku SCXmini diffractometer
Absorption correction: multi-scan (*CrystalClear*; Rigaku, 2005 ▶) *T*_min_ = 0.924, *T*_max_ = 0.924
15436 measured reflections
1886 independent reflections
1452 reflections with *I* > 2σ(*I*)
*R*_int_ = 0.058

#### Refinement

*R*[*F* ^2^ > 2σ(*F* ^2^)] = 0.062
*wR*(*F* ^2^) = 0.165
*S* = 1.12
1886 reflections
91 parameters
H-atom parameters constrained
Δρ_max_ = 0.25 e Å^−3^
Δρ_min_ = −0.54 e Å^−3^

### 

Data collection: *CrystalClear* (Rigaku, 2005 ▶); cell refinement: *CrystalClear*; data reduction: *CrystalClear*; program(s) used to solve structure: *SHELXS97* (Sheldrick, 2008 ▶); program(s) used to refine structure: *SHELXL97* (Sheldrick, 2008 ▶); molecular graphics: *SHELXTL* (Sheldrick, 2008 ▶); software used to prepare material for publication: *PRPKAPPA* (Ferguson, 1999 ▶).

## Supplementary Material

Crystal structure: contains datablocks I, global. DOI: 10.1107/S1600536809035429/im2133sup1.cif

Structure factors: contains datablocks I. DOI: 10.1107/S1600536809035429/im2133Isup2.hkl

Additional supplementary materials:  crystallographic information; 3D view; checkCIF report

## Figures and Tables

**Table 1:** Hydrogen-bond geometry (Å, °)

*D*—H⋯*A*	*D*—H	H⋯*A*	*D*⋯*A*	*D*—H⋯*A*
N1—H1*D*⋯Cl1^i^	0.89	2.47	3.360 (3)	179
N1—H1*E*⋯Cl1^ii^	0.89	2.50	3.209 (2)	137
N1—H1*F*⋯Cl1^iii^	0.89	2.38	3.167 (2)	147

Symmetry codes: (i) 
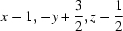
; (ii) 
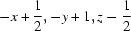
; (iii) 
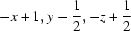
.

**Fig. 1.**
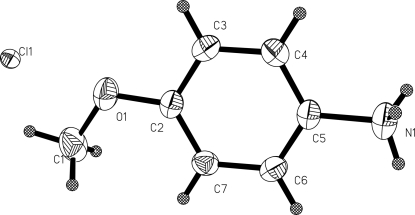
The molecular structure of the title compound showing the atomic numbering scheme. Displacement ellipsoids are drawn at the 30% probability level.

**Fig. 2.**
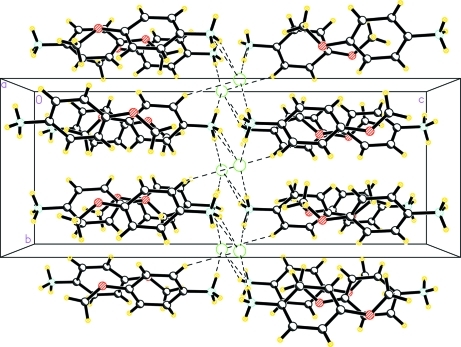
View of the packing of the title compound, stacking along the a axis. Dashed lines indicate hydrogen bonds.

## supplementary crystallographic information

### Comment

Acid-base reactions of organic reactands were already widely researched by ancient chemists (Wu *et al.*, 2006). This study is a part of a systematic investigation of dielectric-ferroelectric materials, including organic ligands, metal-organic coordination compounds and organic-inorganic hybrid materials (Li *et al.*, 2008; Hang *et al.*, 2009). Nevertheless, 4-methoxy-anilinium chloride shows no dielectric irregularity in the temperature range of 80 K to 400 K, (m.p. 401 K).

The asymmetric unit of the title compound is composed of cationic (CH_3_O–C_6_H_4_–NH_3_^+^) and chloride anions (Fig 1). Intramolecular hydrogen bonds between the ammonium groups of the organic cations and the chloride anions are observed in the crystal structure.

### Experimental

Single crystals of 4-methoxy-anilinium chloride are prepared by slow evaporation at room temperature of 20 mL of an ethanolic solution of 4-methoxyphenylamine and an excess of hydrogen chloride (6 mol/L).

### Refinement

All hydrogen atoms were calculated geometrically with C—H distances of 0.93 Å for aromatic C–H functions, 0.96 Å for the methyl group and 0.89 Å for the ammonium substituent. All hydrogen atoms were allowed to ride on the C and N atoms to which they are bonded with thermal parameters of *U*_iso_(H) = 1.2Ueq(parent atom).

### Crystal data

**Table:**

C_7_H_10_NO^+^·Cl^−^	*F*(000) = 672
*M**_r_* = 159.61	*D*_x_ = 1.286 Mg m^−^^3^
Orthorhombic, *P**b**c**a*	Mo *K*α radiation, λ = 0.71073 Å
Hall symbol: -P 2ac 2ab	Cell parameters from 6458 reflections
*a* = 8.905 (2) Å	θ = 3.0–27.6°
*b* = 8.489 (2) Å	µ = 0.40 mm^−^^1^
*c* = 21.817 (4) Å	*T* = 298 K
*V* = 1649.3 (6) Å^3^	Prism, colourless
*Z* = 8	0.20 × 0.20 × 0.20 mm

### Data collection

**Table:**

Rigaku SCXmini diffractometer	1886 independent reflections
Radiation source: fine-focus sealed tube	1452 reflections with *I* > 2σ(*I*)
graphite	*R*_int_ = 0.058
Detector resolution: 13.6612 pixels mm^-1^	θ_max_ = 27.5°, θ_min_ = 3.4°
ω scans	*h* = −11→11
Absorption correction: multi-scan (*CrystalClear*; Rigaku, 2005)	*k* = −10→11
*T*_min_ = 0.924, *T*_max_ = 0.924	*l* = −27→28
15436 measured reflections	

### Refinement

**Table:**

Refinement on *F*^2^	Primary atom site location: structure-invariant direct methods
Least-squares matrix: full	Secondary atom site location: difference Fourier map
*R*[*F*^2^ > 2σ(*F*^2^)] = 0.062	Hydrogen site location: inferred from neighbouring sites
*wR*(*F*^2^) = 0.165	H-atom parameters constrained
*S* = 1.12	*w* = 1/[σ^2^(*F*_o_^2^) + (0.0824*P*)^2^ + 0.5018*P*] where *P* = (*F*_o_^2^ + 2*F*_c_^2^)/3
1886 reflections	(Δ/σ)_max_ < 0.001
91 parameters	Δρ_max_ = 0.25 e Å^−^^3^
0 restraints	Δρ_min_ = −0.53 e Å^−^^3^

### Special details

**Table:**

Geometry. All e.s.d.'s (except the e.s.d. in the dihedral angle between two l.s. planes) are estimated using the full covariance matrix. The cell e.s.d.'s are taken into account individually in the estimation of e.s.d.'s in distances, angles and torsion angles; correlations between e.s.d.'s in cell parameters are only used when they are defined by crystal symmetry. An approximate (isotropic) treatment of cell e.s.d.'s is used for estimating e.s.d.'s involving l.s. planes.
Refinement. Refinement of *F*^2^ against ALL reflections. The weighted *R*-factor *wR* and goodness of fit *S* are based on *F*^2^, conventional *R*-factors *R* are based on *F*, with *F* set to zero for negative *F*^2^. The threshold expression of *F*^2^ > σ(*F*^2^) is used only for calculating *R*-factors(gt) *etc*. and is not relevant to the choice of reflections for refinement. *R*-factors based on *F*^2^ are statistically about twice as large as those based on *F*, and *R*- factors based on ALL data will be even larger.

### Fractional atomic coordinates and isotropic or equivalent isotropic displacement parameters (Å^2^)

**Table:**

	*x*	*y*	*z*	*U*_iso_*/*U*_eq_	
Cl1	0.75138 (6)	0.98509 (7)	0.52111 (3)	0.0470 (3)	
O1	0.1534 (2)	0.1996 (2)	0.29469 (8)	0.0623 (6)	
N1	0.0252 (3)	0.2494 (3)	0.04478 (10)	0.0617 (7)	
H1D	−0.0468	0.3203	0.0383	0.093*	
H1E	−0.0059	0.1553	0.0320	0.093*	
H1F	0.1073	0.2770	0.0242	0.093*	
C2	0.1175 (3)	0.2222 (3)	0.23454 (10)	0.0438 (6)	
C5	0.0595 (3)	0.2423 (3)	0.11049 (11)	0.0441 (6)	
C4	0.1677 (3)	0.1392 (3)	0.13138 (12)	0.0561 (7)	
H4A	0.2203	0.0762	0.1039	0.067*	
C6	−0.0179 (3)	0.3363 (3)	0.15081 (12)	0.0492 (6)	
H6A	−0.0895	0.4069	0.1363	0.059*	
C7	0.0108 (3)	0.3259 (3)	0.21326 (11)	0.0472 (6)	
H7A	−0.0420	0.3890	0.2407	0.057*	
C3	0.1970 (3)	0.1303 (4)	0.19295 (13)	0.0575 (7)	
H3A	0.2710	0.0621	0.2071	0.069*	
C1	0.0870 (4)	0.3025 (4)	0.33900 (13)	0.0761 (10)	
H1A	0.1211	0.2740	0.3792	0.114*	
H1B	−0.0203	0.2934	0.3371	0.114*	
H1C	0.1157	0.4092	0.3304	0.114*	

### Atomic displacement parameters (Å^2^)

**Table:**

	*U*^11^	*U*^22^	*U*^33^	*U*^12^	*U*^13^	*U*^23^
Cl1	0.0461 (4)	0.0472 (4)	0.0478 (4)	−0.0017 (2)	0.0013 (3)	−0.0042 (2)
O1	0.0691 (13)	0.0735 (13)	0.0442 (10)	−0.0047 (11)	−0.0113 (9)	0.0042 (9)
N1	0.0690 (16)	0.0723 (16)	0.0439 (12)	−0.0200 (12)	−0.0091 (11)	0.0082 (11)
C2	0.0435 (14)	0.0466 (12)	0.0414 (13)	−0.0109 (11)	−0.0051 (10)	0.0065 (10)
C5	0.0440 (13)	0.0496 (13)	0.0388 (12)	−0.0143 (11)	−0.0040 (10)	0.0055 (10)
C4	0.0598 (16)	0.0597 (16)	0.0488 (15)	0.0083 (13)	0.0060 (12)	−0.0031 (12)
C6	0.0421 (13)	0.0499 (14)	0.0554 (14)	0.0024 (11)	−0.0056 (11)	0.0060 (12)
C7	0.0404 (13)	0.0526 (15)	0.0486 (13)	−0.0008 (11)	0.0000 (11)	−0.0056 (11)
C3	0.0570 (16)	0.0592 (16)	0.0564 (16)	0.0167 (14)	−0.0053 (13)	0.0062 (13)
C1	0.075 (2)	0.109 (3)	0.0448 (15)	−0.011 (2)	−0.0045 (14)	−0.0147 (16)

### Geometric parameters (Å, °)

**Table:**

O1—C2	1.364 (3)	C4—C3	1.370 (4)
O1—C1	1.431 (4)	C4—H4A	0.9300
N1—C5	1.467 (3)	C6—C7	1.389 (4)
N1—H1D	0.8900	C6—H6A	0.9300
N1—H1E	0.8900	C7—H7A	0.9300
N1—H1F	0.8900	C3—H3A	0.9300
C2—C7	1.376 (4)	C1—H1A	0.9600
C2—C3	1.390 (4)	C1—H1B	0.9600
C5—C6	1.373 (4)	C1—H1C	0.9600
C5—C4	1.380 (4)		
			
C2—O1—C1	117.9 (2)	C5—C6—C7	119.9 (2)
C5—N1—H1D	109.5	C5—C6—H6A	120.0
C5—N1—H1E	109.5	C7—C6—H6A	120.0
H1D—N1—H1E	109.5	C2—C7—C6	119.9 (2)
C5—N1—H1F	109.5	C2—C7—H7A	120.0
H1D—N1—H1F	109.5	C6—C7—H7A	120.0
H1E—N1—H1F	109.5	C4—C3—C2	120.8 (2)
O1—C2—C7	125.2 (2)	C4—C3—H3A	119.6
O1—C2—C3	115.4 (2)	C2—C3—H3A	119.6
C7—C2—C3	119.4 (2)	O1—C1—H1A	109.5
C6—C5—C4	120.5 (2)	O1—C1—H1B	109.5
C6—C5—N1	119.8 (2)	H1A—C1—H1B	109.5
C4—C5—N1	119.6 (2)	O1—C1—H1C	109.5
C3—C4—C5	119.5 (2)	H1A—C1—H1C	109.5
C3—C4—H4A	120.3	H1B—C1—H1C	109.5
C5—C4—H4A	120.3		
			
C1—O1—C2—C7	−6.9 (4)	O1—C2—C7—C6	−178.8 (2)
C1—O1—C2—C3	173.4 (3)	C3—C2—C7—C6	0.9 (4)
C6—C5—C4—C3	0.4 (4)	C5—C6—C7—C2	0.4 (4)
N1—C5—C4—C3	−178.5 (2)	C5—C4—C3—C2	1.0 (4)
C4—C5—C6—C7	−1.1 (4)	O1—C2—C3—C4	178.1 (3)
N1—C5—C6—C7	177.8 (2)	C7—C2—C3—C4	−1.6 (4)

### Hydrogen-bond geometry (Å, °)

**Table:**

*D*—H···*A*	*D*—H	H···*A*	*D*···*A*	*D*—H···*A*
N1—H1D···Cl1^i^	0.89	2.47	3.360 (3)	179
N1—H1E···Cl1^ii^	0.89	2.50	3.209 (2)	137
N1—H1F···Cl1^iii^	0.89	2.38	3.167 (2)	147

Symmetry codes: (i) *x*−1, −*y*+3/2, *z*−1/2; (ii) −*x*+1/2, −*y*+1, *z*−1/2; (iii) −*x*+1, *y*−1/2, −*z*+1/2.
